# A pond-side test for Guinea worm: Development of a loop-mediated isothermal amplification (LAMP) assay for detection of *Dracunculus medinensis*

**DOI:** 10.1016/j.exppara.2020.107960

**Published:** 2020-10

**Authors:** Neil Boonham, Jenny Tomlinson, Sioban Ostoja-Starzewska, Robbie A. McDonald

**Affiliations:** a Newcastle University, Kings Rd, Newcastle Upon Tyne, NE1 7RU, UK; b Fera Science Ltd, Sand Hutton, York, YO41 1L, UK; c Environment and Sustainability Institute, University of Exeter, Penryn, TR10 9FE, UK

**Keywords:** Diagnostics, LAMP, Guinea worm, *Dracunculus medinensis*

## Abstract

Guinea worm *Dracunculus medinensis* causes debilitating disease in people and is subject to an ongoing global eradication programme. Research and controls are constrained by a lack of diagnostic tools. We developed a specific and sensitive LAMP method for detecting *D. medinensis* larval DNA in copepod vectors. We were able to detect a single larva in a background of field-collected copepods. This method could form the basis of a “pond-side test” for detecting potential sources of Guinea worm infection in the environment, in copepods, including in the guts of fish as potential transport hosts, enabling research, surveillance and targeting of control measures. The key constraint on the utility of this assay as a field diagnostic, is a lack of knowledge of variation in the temporal and spatial distribution of *D. medinensis* larvae in copepods in water bodies in the affected areas and how best to sample copepods to obtain a reliable diagnostic sample. These fundamental knowledge gaps could readily be addressed with field collections of samples across areas experiencing a range of worm infection frequencies, coupled with field and laboratory analyses using LAMP and PCR.

## Highlights

• LAMP tests were developed for *Dracunculus medinensis* and *D. insignis* and were shown to be specific and sensitive.
• A LAMP test was developed to amplify DNA from copepods to use as an internal amplification control during testing.
• Samples of copepods taken from ponds could be tested using the LAMP tests and *Dracunculus medinensis* could be detected.
• Results are achieved in less than 30 min using just the Genie III instrument and no other laboratory equipment is necessary.

## 1 Introduction

Guinea worm *Dracunculus medinensis* is a nematode parasite that causes profoundly debilitating disease, dracunculiasis, in people. The worm has been subject to a global eradication campaign since the 1980s, when incidence was estimated to be around 3.5 million cases per annum (Watts, 1987). The campaign has been immensely successful and has reduced numbers of human cases to only 28 in 2018 (Centers for Disease Control, 2019). The location of cases has also been limited to very few countries; Chad, Ethiopia, Mali and South Sudan are considered endemic, of which Chad is the worst affected, while Cameroon and Angola have experienced a small number of recent cases. The worm looked likely to be only the second human pathogen, after smallpox virus, to be eradicated. The eradication campaign, however, has experienced a major setback with the apparent emergence of non-human animal reservoirs, principally in domestic dogs *Canis familiaris* (Eberhard et al., 2014; McDonald et al., 2020)*.* There were 1040 infections in dogs in Chad in 2018 and dog infections have also been recorded in Ethiopia and Mali. The worms extracted from dogs and people are genetically indistinguishable (Thiele et al., 2018). The existing range of control measures, principally surveillance, detection and isolation of cases, and treatment of water with organophosphates, although immensely successful with people, have not yet turned the tide on rising counts of infections in animals. Therefore, new tools to identify and then to treat environmental sources of infection will add to the hitherto effective, albeit limited, repertoire of control measures.

The obligate vectors of *D. medinensis* are freshwater Crustacea of the copepod family Cyclopidae, including *Mesocyclops*, *Metacyclops, Thermocyclops* and others (Cairncross et al., 2002). The parasite is classically transmitted via drinking water carrying infected copepods. However, there is the possibility of alternative pathways, via fish or frogs acting as paratenic or transport hosts (Eberhard et al., 2014). Irrespective of the pathway, a key tool for control is the treatment of water bodies, that represent a potential or actual source of infection, with organophosphates (temephos, Abate), which will likely remain a mainstay of Guinea worm control and eradication campaigns.

For adult worms, there is no pre-patent diagnostic in the live host and worm infection can only be diagnosed when the adult female emerges. For larvae in copepods, microscopic diagnosis is the standard approach, though this is extremely labour intensive. Therefore, a rapid diagnostic approach that could be deployed, ideally in field settings, i.e. a “pond-side test” that could sensitively and specifically identify *D. medinensis* DNA in complex matrices of mixed species of freshwater invertebrates sampled from ponds, or from fish or frog guts and tissues, would therefore potentially improve epidemiological understanding of the infection and its distribution in the environment and ideally increase the efficacy and efficiency of labour-intensive control measures. Such a test would also have potential application as a research tool to address the major knowledge-gap in relation to the distribution of *D. medinensis* in the environment, since virtually nothing is known about temporal and spatial variation in sources of *D. medinensis* infection. In due course, a test might also be used as part of surveillance programmes oriented towards certification of disease elimination.

Diagnostics based on DNA amplification are commonplace in laboratories and are used for the sensitive and specific detection of a range of pathogens, pests and parasites. Predominantly based on the polymerase chain reaction (PCR) they generally require well equipped laboratories with capacity to extract and purify DNA from samples followed by amplification on laboratory instruments capable of accurate thermal-cycling. In recent years the development of isothermal DNA amplification approaches (amplification at a single temperature) such as Loop mediated AMPlification (LAMP) provide the potential for achieving the same levels of analytical precision in remote and under-resourced locations, including directly in the field for the detection of parasites (e.g. Tong et al., 2015; Cook et al., 2015; Lodh et al., 2017; Fallahi et al., 2018). No thermal cycling is required, enabling the use of simple, battery powered, portable equipment such as the Genie III (Optisense, Horsham, UK) and the enzymes used are sufficiently robust that DNA template does not need to be purified to achieve amplification, something that is not possible using PCR methods. The objective of the current work was to develop the basis of a pond-side detection method for *D. medinensis*, suited for use in remote and poorly-resourced locations and that could be used for parasite detection in copepods sampled from ponds, or other similarly complex matrices, such as fish guts containing copepods and their remains. The overall aim is to improve understanding of Guinea worm epidemiology and to enable targeting of ponds for organophosphate treatment, as part of the Guinea worm eradication programme.

## 2 Methods and results

LAMP primers (Table 1) for *D. medinensis* and the closely related but allopatric *D. insignis* were designed to the cytochrome oxidase I gene (COI). The primers targeted conserved regions that were specific for the species but were divergent for the non-target species including *D. lutrae* and sequences from other genera with the most similar COI sequences available from GenBank. LAMP primers were designed to the 18S rRNA gene, targeting conserved regions of sequence from copepods within genera including *Cyclops, Eucyclops* and *Mesocyclops*. LAMP reactions were run by incubating samples for 30 min at 62 °C on the Genie III instrument with data collection in real-time. Assays were run using Isothermal MasterMix (ISO004 from OptiGene, Camberley, UK) containing an DNA intercalating fluorescent dye and primers at the following final concentrations F3/B3 = 0.2 μM, FIP/BIP = 2 μM and F-LOOP/B-LOOP = 1 μM. Real-time LAMP results were recorded as the time to reach a positive result (Tp) and an annealing temperatures (Ta), indicating the specific temperature at which the amplification product is annealed.

**Table 1:** Primer sequences for the LAMP assays specific for *Dracunculus medinensis*, *D. insignis* and the copepod internal positive control.

Primer	Sequence (5′–3′)
D.med F3	GTTATTACTTCTCATGCTATTATGATA
D.med B3	AAACTAAAAATAGCCAAATCAACTCTAT
D.med FIP	ACAGGCATCAATCAATAACTAACATTATTCTGGTAATGCCTAGTTTGATTGGA
D.med BIP	AATATTGATTTTGTCTGCTTGTTTGGTGCCAGGATGACCCCTAGTACTT
D.med F-loop	ATATCAGGAGCCCCCAACATT
D.med B-loop	TCTTGTGGAACTAGATGGACTA
D.insig F3	TTTTTATGGTTATGCCTAGTTTGATT
D.insig B3	CAGAACAATGTAAACTAAAAATAGCCA
D.insig FIP	CATCAAAGAAACAGGTATTAACCAATAACGGTGGTTTTGGTAATTGGATAGTT
D.insig BIP	AGTCGATAGTTCTTGTGGTACTAGTTCAACACTATTTCCAGGATGACCA
D.insig F-loop	CACATTATTCAAACGAGGAAAACTT
D.insig B-loop	GAACTGTTTATCCTCCTTTGAGT
Copepod F3	GTCGACTGTGGCATAGACG
Copepod B3	CTCATTCCGATTACCAGGCC
Copepod FIP	TCAGGCTCCCTCTCCGGAATCCCACAGTGGTTTTGACGG
Copepod BIP	AAGGCAGCAGGCACGCAAATTCGGATGAGTCTGGTATCGT
Copepod F-loop	CGAACCCTAATTCCCCGTTAC
Copepod B-loop	GCCGAGGTAGTGACGAAAAAT

DNA was extracted from an adult specimen of *D. medinensis* using a DNeasy extraction kit following the manufacturers protocols (Qiagen). The DNA concentration was estimated using a NanoDrop following the manufacturers recommended protocols (ThermoFisher) before being serially diluted and tested using the *D. medinensis* LAMP assay. The lowest dilution that was routinely detected by the assay was observed to be 100 fg DNA. Inclusivity testing was performed using 25 DNA extracts from specimens of *D. medinensis* obtained from four hosts (dog, human, domestic cat *Felis catus* and olive baboon *Papio anubis*) from Chad, Ethiopia, Mali and South Sudan (Table 2). The results show that all samples gave a positive result and no cross reactivity with *D. insignis* or DNA from uninfected copepod was observed. The limit of detection of the *D. insignis* assay was equivalent to that of the *D. medinensis* assay, and no cross reactivity was observed with DNA extracted from *D. medinensis* or uninfected copepods (data not shown).

**Table 2:** Real-time LAMP results for DNA extraction from *Dracunculus medinensis* specimens from a range of host species and countries of origin. – indicates no detection and n/a indicates not applicable.

Extract ID	Origin	Host	*Dracunculus medinensis* assay	
			Time to positive (min:sec)	Annealing temperature (°C)
ChDo-339.2	Chad	Dog	10:00	81.11
ChHu-69	Chad	Human	12:15	80.55
ChDo-1	Chad	Dog	7:45	81.34
ChDo-108.5	Chad	Dog	8:15	81.30
ChHu-25	Chad	Human	8:45	80.86
ChCa-10a	Chad	Cat	9:00	81.15
ChDo-189	Chad	Dog	10:15	81.50
ChDo-13	Chad	Dog	8:00	81.40
ChDo-11	Chad	Dog	9:00	80.86
ChDo-47.11	Chad	Dog	8:30	81.60
ChHu-100	Chad	Human	10:15	81.35
ChHu-24	Chad	Human	8:30	81.45
ChDo-1	Chad	Dog	7:45	81.20
ChHu-283	Chad	Human	10:00	82.58
EtDo-172	Ethiopia	Dog	12:15	82.78
EtHu-278	Ethiopia	Human	10:30	82.63
EtBa-153	Ethiopia	Baboon	10:30	82.87
EtHu-152	Ethiopia	Human	10:00	82.57
MaHu-165	Mali	Human	9:45	82.67
MaHu-240	Mali	Human	10:00	82.54
MaHu-242	Mali	Human	23:00	82.58
MaHu-253	Mali	Human	11:00	82.79
SsHu-201	South Sudan	Human	8:45	82.50
SsHu-256	South Sudan	Human	10:15	82.36
SsDo-183	South Sudan	Dog	10:30	82.48
Uninfected copepod	n/a	n/a	–	–
*Dracunculus insignis*	n/a	n/a	–	–

Using copepods of unknown genera collected from ponds in villages alongside the River Chari in Chad, where Guinea worm infections in dogs were commonplace (McDonald et al., 2020), DNA was extracted using several methods, to identify the one most suited to pond-side use. As a standard, DNA was extracted from single copepods using a DNeasy extraction kit following the manufacturers protocols (Qiagen). The DNA concentration was estimated using a NanoDrop (ThermoFisher) then serially diluted and tested using the copepod LAMP assay. The limit of detection of the copepod LAMP assay was observed to be similar to that of the *Dracunculus* assays (<100 fg), and no cross reactivity was observed with DNA extracted from *D. medinensis* or *D. insignis*. DNA was also extracted by homogenising copepods using a plastic micropestle in a microcentrifuge tube containing 50 μl nuclease-free water. The homogenate (1 μl) was tested directly using the copepod LAMP test and amplification was observed between 7 and 25 min. Individual copepods were incubated in 50 μl 0.6 M KOH to disrupt the samples. The resulting solution was tested directly using the copepod LAMP test using isothermal master mix specifically buffered for use with alkaline samples (Lyse & LAMP - Optigene). Using this method, amplification was observed between 4 and 9 min.

The alkaline lysis extraction method was used to test samples containing individual *Dracunculus* larvae spiked into pools of cyclopoid copepods known to not contain *Dracunculus*. Samples containing different numbers of individual copepods and either a single *D. insignis* L1 or a single *D. medinensis* L3 were prepared. DNA was extracted from each sample using the alkaline lysis method and tested using LAMP. The results (Table 3) shows that single larvae could be detected in a background containing 30 copepods. The efficiency of the control amplification was unaffected when larger numbers (>100) of copepods were extracted and tested.

**Table 3:** Summary of real-time LAMP results testing samples comprising *Dracunculus*-free copepods spiked with *D. medinensis* or *D. insignis* larvae. Nt = sample not tested, - = negative result. -ve sample is a no-template control, +ve controls are copepods mixed with *Dracunculus* DNA. Tp = time to positive, Ta = Annealing temperature.

Number spiked into sample			Samples tested with LAMP assays for					
Copepods	*D. medinensis*	*D. insignis*	*D. medinensis*		*D. insignis*		Copepod	
			Tp (min:sec)	Ta (°C)	Tp (min:sec)	Ta (°C)	Tp (min:sec)	Ta (°C)
1	1	0	8:15	82.20	nt	nt	6:30	87.49
10	1	0	7:45	82.07	nt	nt	6:15	87.65
20	1	0	8:00	82.09	nt	nt	5:15	87.78
30	1	0	8:15	82.04	nt	nt	5:30	87.79
30	1	0	8:45	81.97	nt	nt	5:30	87.76
10	0	0	–	–	nt	nt	6:30	87.21
20	0	0	–	–	nt	nt	6:00	87.75
≥50	0	0	–	–	nt	nt	6:15	87.16
≥100	0	0	–	–	nt	nt	6:15	87.21
1	0	1	nt	nt	10:15	82.50	9:00	88.26
10	0	1	nt	nt	13:00	82.49	7:30	88.31
10	0	0	nt	nt	–	–	7:00	88.26
-ve control			–	–	–	–	–	–
	+ve control		7:30	81.83	nt	nt	6:00	87.67
		+ve control	nt	nt	11:30	82.42	8:00	88.41

Copepods were isolated from pond water samples collected in Chad. DNA was extracted from individual and groups of copepods and tested using the LAMP assays. Of the 208 samples tested (38 individual, 13 bulks of 10 copepods and 2 bulks of 20 copepods) only one sample (tested individually) gave a positive result with a Tp of approximately 14 min.

## 3 Discussion

The methods developed here bring the potential ability to identify *D. medinensis* in samples taken from water sources, using technology that could be used either “pond-side” or in rudimentary laboratory environments with problematic power supplies. This would allow rapid, unambiguous detection of contaminated sources in order to target the control of copepods using temephos (Abate) in the water bodies containing infected copepods. Given the conspicuous lack of knowledge of the distribution of *D. medinensis* larvae in water sources, even in areas known to be badly affected by Guinea worm infections, and which represent the major challenges for global eradication of this human disease, this tool also has potential application in research towards better understanding of disease epidemiology.

Our LAMP assay has been shown to be sensitive and specific in laboratory conditions and to varying the availability and presentation of the target DNA. Some further validation of how the assay might operate in field conditions, is clearly required, particularly in respect of taxonomically diverse, numerically abundant samples of freshwater invertebrates. However, a key unknown is the distribution of *D. medinensis* in the field. Surprisingly, given the success of the control programmes over a period of >30 years, there remains next to no knowledge of how the pathogen is distributed among water sources, within water sources or among host species and individuals, and how these distributions might vary with time or space. It is likely that the infection is patchy in both time and space. Therefore, the utility of this assay in a control campaign depends primarily, not on the performance of the assay itself, but on the ability of the field copepod sampling regime to reliably extract infected material from the environment when it is truly there. Therefore, a programme of validation of the field sampling protocol, using this assay and other standards, perhaps including laboratory-based PCR, is required.

## CRediT authorship contribution statement

**Neil Boonham:** Conceptualization, Funding acquisition, Investigation, Project administration, Writing - original draft, review & editing. **Jenny Tomlinson:** Investigation, Methodology, Validation, Visualization, review & editing. **Sioban Ostoja-Starzewska:** Investigation, Methodology, Validation, Visualization, review & editing. **Robbie A. McDonald:** Conceptualization, Funding acquisition, Project administration, Validation, Visualization, review & editing.
